# Anthrax outbreak associated with the consumption and handling of carcasses of livestock that suddenly died, Kanungu District, Uganda, June–November 2024

**DOI:** 10.1371/journal.pntd.0013727

**Published:** 2026-07-10

**Authors:** Charity Mutesi, Richard Migisha, Hannington Katumba, Bridget Ainembabazi, Helen Nelly Naiga, Scott Kellermann, Birungi Mutahunga, Fred Ouma, Aloysius Tumwesigye, Frank Ilimaso, Lilian Bulage, Alex Riolexus Ario

**Affiliations:** 1 Uganda Public Health Fellowship Program, Uganda National Institute of Public Health, Kampala, Uganda; 2 Bwindi Community Hospital, Medical Services Department, Buhoma, Kanungu District, Uganda; 3 Kanungu District Local Government, District Health Office, Kanungu District, Uganda; Colorado State University, UNITED STATES OF AMERICA

## Abstract

**Background:**

Anthrax is a recurrent zoonosis in Uganda, with 11 outbreaks reported in 2024. On September 17, 2024, the Ministry of Health received reports of two human deaths in Kanungu District, later confirmed as anthrax—the district’s first recorded outbreak. We investigated to determine its scope, risk factors, and recommend control measures.

**Methods:**

We defined a suspected cutaneous anthrax case as an acute onset of skin lesions (papule, vesicle, or eschar) with ≥2 of itching, reddening, lymphadenopathy, fever, or malaise. Suspected gastrointestinal anthrax was onset of abdominal pain with ≥2 of vomiting, diarrhea, fever, or loss of appetite in a Kanungu resident from June–November 2024. Confirmation required Polymerase Chain Reaction (PCR) detection of *Bacillus anthracis*. In a 1:2 unmatched case-control study, we enrolled all cases and controls from neighboring asymptomatic households in the two most affected sub-counties. Logistic regression was used to identify risk factors for infection.

**Results:**

We identified 90 cases (86 suspected and 4 confirmed); 80% had cutaneous, 11% gastrointestinal, and 9% both forms. Males were more affected (attack rate [AR]=48/100,000) than females (AR = 15/100,000); case fatality rate was 6.7% (6/90). Overall, 10/27 sub-counties were affected, with Bugongi (AR = 257/100,000) being the most affected. Consuming meat from suddenly dead livestock (adjusted odds ratio [aOR]=5.5, 95% CI: 2.7–11), handling carcasses (aOR=8.2, 95% CI: 3.8–18), having a low education level (aOR=6.0, 95% CI: 2.6–14), and being male (aOR=2.3, 95%CI = 1.03-4.9) increased the odds of infection. Notably, 90% of the case-patients purchased the contaminated meat from a single dealer.

**Conclusion:**

The outbreak was linked to consuming and handling meat from livestock that died suddenly, underscoring gaps in veterinary inspection and carcass management. Strengthening inspections, enforcing carcass disposal, and targeted community education are essential for prevention.

## Author summary

Anthrax is a recurring zoonotic disease in Uganda, but some districts remain unaffected until a first outbreak occurs. In September 2024, an unusual cluster of human deaths in Kanungu District was confirmed as anthrax, prompting an investigation to understand how the outbreak spread and who was most at risk. The investigation identified 90 cases, with most patients presenting with the cutaneous form of the disease, and a smaller proportion experiencing gastrointestinal symptoms or both. Males were more affected than females, and the outbreak extended across multiple sub-counties, with certain areas experiencing higher attack rates. The study found that the infection was strongly associated with consuming meat from animals that had died suddenly and handling contaminated carcasses. Lower levels of education and being male also increased the likelihood of infection. Notably, most affected individuals had obtained meat from a single source, suggesting a common exposure point. These findings indicate that the outbreak was driven by unsafe handling and consumption of contaminated meat, reflecting gaps in veterinary oversight and carcass disposal practices. Strengthening meat inspection systems, enforcing safe disposal of animal carcasses, and improving community awareness are critical steps to prevent similar outbreaks in the future.

## Background

Anthrax is an acute zoonotic disease caused by the gram-positive, spore-forming bacterium *Bacillus anthracis*. Although cases occur worldwide, outbreaks are more common in low- and middle-income countries than in developed regions [1–6]. The pathogen’s spores, which can survive for decades in soil at former burial sites of infected animals, have sparked new infections even several decades after site disturbance [7,8]. Human transmission typically follows direct contact with infected animals or consumption of contaminated meat, but can also occur through inhalation of spores or handling animal products such as hair, wool, hides, or bone and their derivatives such as drums, brushes, and rugs [9,10].

Anthrax presents in four clinical forms, each with distinct transmission routes and incubation periods [11–13]. Cutaneous anthrax, the most common, manifests as skin lesions at sites of spore entry, typically appearing 1–12 days after exposure (average 5–7 days) [14]. Gastrointestinal anthrax arises from ingesting undercooked meat contaminated with *B. anthracis*, with symptoms developing within 2–5 days [15]. Inhalational anthrax, the most severe form, follows inhalation of spores from contaminated animal products (e.g., hides or wool) and may incubate anywhere from 4 to 6 days [16–18]. Finally, injection anthrax is transmitted by intravenous, subcutaneous or intramuscular injection of contaminated heroin. Its incubation periods are estimated from a day or less to 10 days or more [19]. It’s pertinent to note that a single animal case within a community can precipitate wider human and animal outbreaks unless promptly and effectively contained [2,20–22].

Anthrax is one of the seven priority zoonotic diseases in Uganda [23]. In Uganda, recurrent anthrax outbreaks are often associated with handling or consumption of meat from animals found dead [4,24]. Between January 2017 and April 2022, the country recorded 19 outbreaks; seven in the western region, and an additional eleven outbreaks occurred during 2024, three of which affected western districts [1,2,4,24,25].

On September 17, 2024, the Ministry of Health received reports of two human deaths from laboratory-confirmed anthrax in Kanungu District. This coincided with reports of unexplained livestock deaths on multiple local farms in the district, which exhibited classic anthrax signs. We investigated the outbreak to: (1) describe the magnitude and epidemiologic characteristics of anthrax cases in Kanungu District, Uganda, during June–November 2024; (2) identify risk factors associated with illness; and (3) recommend evidence-based control and prevention measures.

## Methods

### Ethics statement

This outbreak investigation was in response to a public health emergency and was therefore determined to be non-research. The Ministry of Health (MoH) permitted the investigation of this outbreak. In agreement with the International Guidelines for Ethical Review of Epidemiological Studies by the Council for International Organizations of Medical Sciences (1991) and the Office of the Associate Director for Science, United States Center for Disease Control (US CDC) and Uganda, it was determined that this activity was not human subject research and that its primary intent was public health practice or disease control activity (specifically, epidemic or endemic disease control activity). This activity was reviewed by the US CDC and was conducted consistent with applicable federal law and CDC policy. §§See, e.g., 45 C.F.R. part 46, 21 C.F.R. part 56; 42 U.S.C. §241(d); 5 U.S.C. §552a; 44 U.S.C. §3501 et seq. All experimental protocols were approved by the US CDC human subjects review board (The National Institute for Occupational Safety and Health Institutional Review Board) and the Uganda MoH and were performed in accordance with the Declaration of Helsinki. Permission to conduct the outbreak response was also granted by Kanungu District Local Government. Before data collection, informed verbal consent was obtained from all the participants who were aged 18 years or older (legal age in Uganda). For those below 18 years, verbal consent was sought from their parents/guardians, and assent was also obtained from them to participate in the study.

### Outbreak setting

The outbreak occurred in Kanungu District, located in the pastoral lands of southwestern Uganda (S1 Fig). Administratively, Kanungu comprises 27 sub-counties, with a population of approximately 293,537 according to the 2022 census [26]. The District is bordered by Rukungiri District to the north and east, Rubanda District to the south-east, Kisoro District to the south-west, and the Democratic Republic of Congo to the west. Approximately 85% of households in Kanungu District are dependent on subsistence farming as a main source of livelihood, with approximately 80% engaged in rearing livestock [27]. This was the first-time anthrax was reported in the district, where almost half of it was affected.

### Case definition and case finding

The investigation period was defined as June 1–November 30, 2024. June 1, 2024, was selected as the start date to allow identification of potential earlier cases before the introduction of the suspected infected livestock and the earliest known exposures reported during the investigation. November 30, 2024, was selected as the end date to include the last identified case during prospective case finding and to allow sufficient follow-up time after implementation of control measures.

We defined a suspected cutaneous anthrax case as an acute onset of skin lesions (papule or vesicle or eschar) with ≥2 of the following symptoms: skin itching, skin reddening, lymphadenopathy, headache, fever, loss of appetite, and general body weakness in a resident of Kanungu District, from June 1, 2024–November 30, 2024.

A suspected gastrointestinal anthrax case was defined as the onset of abdominal pain with ≥2 of the following symptoms: vomiting, diarrhea, swelling of lymph nodes, fever, general body weakness, loss of appetite, headache, and pain on swallowing in a resident of Kanungu District, from June 1, 2024–November 30, 2024.

We defined a confirmed anthrax case as a suspected case with a clinical sample (skin lesion or blood) testing positive for *B. anthracis* by polymerase chain reaction (PCR).

To identify cases, we visited and reviewed medical records at public and private health facilities in the affected sub-counties. With the help of community health workers, we conducted an active case search in the community and updated the line list. In addition, we collected information on demographic characteristics and symptom presentation.

### Descriptive epidemiology

We described cases by person, place, and time. Using the 2022 projected population data for sub-counties, we computed attack rates (AR) by age, sex, and sub-county and constructed maps depicting the cases’ residences. We used epidemiological curves to describe the course of the outbreak in humans over time.

### Laboratory analysis

In humans, whole blood and swab samples were obtained from anthrax-suspected cases. In livestock, swabs and tissue samples (ear lobe) were collected from carcasses of animals that recently died from suspected anthrax and had not been euthanized for routine slaughter. When available, samples were collected directly from intact carcasses before disposal. Soil samples were collected from areas suspected to be contaminated with Bacillus anthracis spores. These included soil surrounding locations where animal carcasses had been slaughtered, butchered, or disposed of. Approximately 5–10 grams of soil were collected from the top 2–5 cm of the soil surface at each sampling site using sterile scoops. Samples were placed in sterile containers and labeled appropriately. All samples were packaged using a triple package technique and transported through the national hub system.

The hub system is a coordinated laboratory network for the collection, transportation, and testing of samples, designed to ensure efficient and secure movement across different parts of the country for timely analysis and diagnosis. Samples were sent to the Uganda Virus Research Institute (UVRI), Arua, for anthrax testing in humans, and the National Animal Disease Diagnostics and Epidemiology Centre (NADDEC) laboratory in Entebbe, Uganda, for anthrax testing in livestock.

### Environmental assessment

We inspected animal farms in the affected villages using snowballing and house-to-house visits to identify farms that had reported sudden deaths of cattle, goats, or sheep within the ten affected sub-counties between June and November 2024. This was done to identify the source of the anthrax outbreak in Kanungu District and also determine how the implicated meat was distributed.

We interviewed the farm owners and herdsmen to gather information on the farm management practices and how the meat and other animal products were distributed. We visited the affected farms and observed the pasture in the grazing area, the water drinking areas for livestock, and where the livestock were slaughtered or buried. We also interviewed identified dealers of meat from livestock that died suddenly and were slaughtered for distribution to understand the distribution chain of the implicated meat. Environmental samples were not collected from the premises of meat dealers.

### Hypothesis generation interviews

We conducted interviews with the 20 suspected cases to identify possible sources and factors associated with contracting anthrax. Based on these interviews, we hypothesized that handling and consuming meat from livestock that died suddenly was associated with developing anthrax. We also explored other risk factors, including educational level and occupation of the cases.

### Case-control study

We conducted an unmatched case-control study in the most affected sub-counties of Katete and Bugongi. An unmatched design was selected to allow flexibility in analysis and to facilitate rapid implementation of prevention and control measures during the outbreak investigation. Controls were selected at a 1:2 ratio from the same village as cases ensure comparability with respect to community-level and environmental exposures. For each case, two neighboring households were identified. In each selected household, all eligible members present at the time of data collection were listed, and one individual was selected using simple random sampling (lottery method), whereby names were written on separate pieces of paper and one was drawn blindly. We used a structured questionnaire to collect demographic data, including age, sex, place of residence, and occupation, as well as data on potential exposures (eating meat from livestock that had died suddenly, handling suddenly dead livestock, and preparation methods). We used logistic regression to identify factors associated with anthrax. Variables that had a p-value <0.2 at the bivariate level were included in the final model for multivariable analysis, and corresponding adjusted odds ratios (aOR) and 95% confidence intervals were reported. We further conducted a common reference group analysis to assess the effect of the exposures [28].

## Results

### Descriptive epidemiology

We identified a total of 90 cases (86 suspects and 4 confirmed cases). Males were more affected with an attack rate (AR) of 48/ 100,000 compared to females with an AR of 15/ 100,000. The mean age of cases was 37 years [Standard Deviation (SD) ± 14 (range 6–78)]. Cases aged 40–49 years were most affected (AR = 132/100,000) followed by those that were aged 30–39 years (AR = 74/ 100,000). A case fatality rate of 6.7% (6/90) was noted in the outbreak (Table 1).

**Table 1:** Attack rates by sex and age among cases during the anthrax outbreak in Kanungu District, June–November 2024.

Characteristic	Cases (n = 90)	Population	AR/100,000
**Gender**			
Male	68	142,000	48
Female	22	151,200	15
**Age group**			
0-9	2	85520	2
10-19	9	76117	12
20-29	14	51585	27
30-39	23	31285	74
40-49	28	21167	132
50-59	9	13772	65
60-69	3	8213	37
70-79	2	4015	50

Case fatality rate: 6.7%; AR: Attack rate.

### Clinical symptoms of cases by the type of anthrax during the anthrax outbreak in Kanungu District, June–November 2024

**Fig 1:**
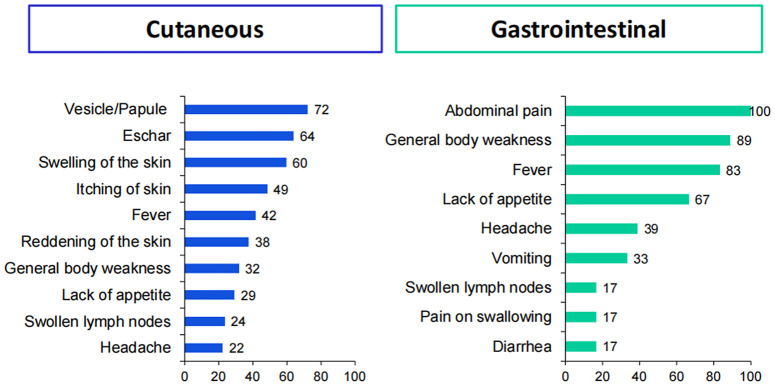
Clinical signs and symptoms reported among suspected and confirmed human anthrax case-patients (N = 90) during an outbreak investigation in Kanungu District, southwestern Uganda, June–November 2024.

Of the 90 cases, 72 (80%) presented with signs and symptoms suggestive of cutaneous anthrax, 10 (11%) had only gastrointestinal anthrax, and 8 (9%) had a combination of both cutaneous and gastrointestinal anthrax. Most cutaneous anthrax cases presented with swelling and itching of the skin in addition to skin lesions, while most gastrointestinal cases presented with general body weakness, followed by fever and loss of appetite (Fig 1).

### Distribution of anthrax cases by date of symptom onset

**Fig 2:**
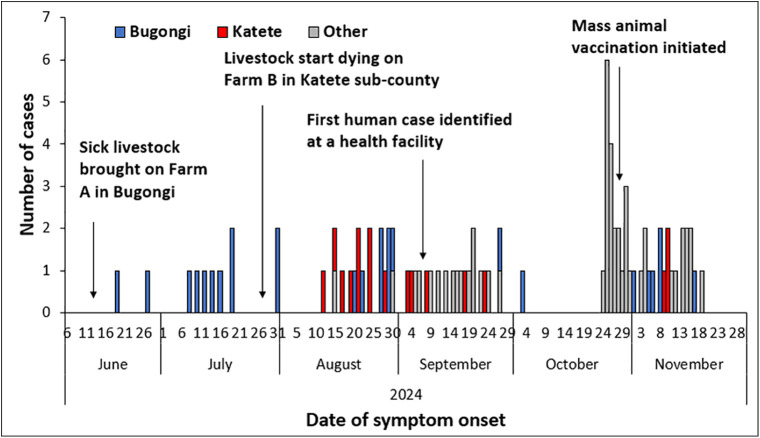
Distribution of human anthrax cases (N = 90) by sub-county of residence in Kanungu District, southwestern Uganda, June–November 2024.

On June 14, 2024, sick livestock from a neighboring district were introduced to a private farm (Farm A) in Bugongi Sub-county. This was followed by multiple deaths of these animals on the farm the following day. The carcasses were butchered and distributed within the community. The earliest known human case following our case investigation developed symptoms on June 24, 2024, after participating in the butchering of the contaminated carcasses and meat distribution. However, he sought care at a local drug shop and was not detected through the district surveillance system. Additional livestock deaths in the affected sub-counties were reported to animal health workers but were clinically misdiagnosed as Black Quarter (BQ), and BQ vaccination was administered. Despite vaccination, animal deaths persisted. Human cases continued to emerge starting from sub-counties where the affected farms were located but remained undetected by the surveillance system. On July 25, livestock started dying on Farm B, which neighbored Farm A. On September 9, 2024, a suspected human anthrax case reported to a health facility, which notified the District Health Office. Eight days later, on 17th September 2024, the Ministry of Health received reports from the district about two human deaths confirmed with anthrax, triggering the outbreak investigation. It took 83 days to detect this anthrax outbreak in humans. To identify earlier and additional cases, we conducted both retrospective and prospective active case searches in affected communities and healthcare facilities. Following the outbreak, mass animal vaccination was initiated on 25th October, together with other control measures, such as safe carcass disposal (Fig 2).

### Laboratory investigation findings

A total of 63 (18 human, 26 animal, and 15 soil) samples were collected and tested for Bacillus anthracis. Not all identified cases were sampled because many had already received antibiotics before investigation, had healed lesions, or were identified retrospectively during active case finding in the community, limiting the feasibility of specimen collection. Four of the human samples returned PCR positive (positivity rate) of 22%) while four animal samples tested positive for anthrax. Soil samples (n = 15) collected from affected farms and slaughter sites tested negative for Bacillus anthracis.

### Environmental assessment findings

**Fig 3:**
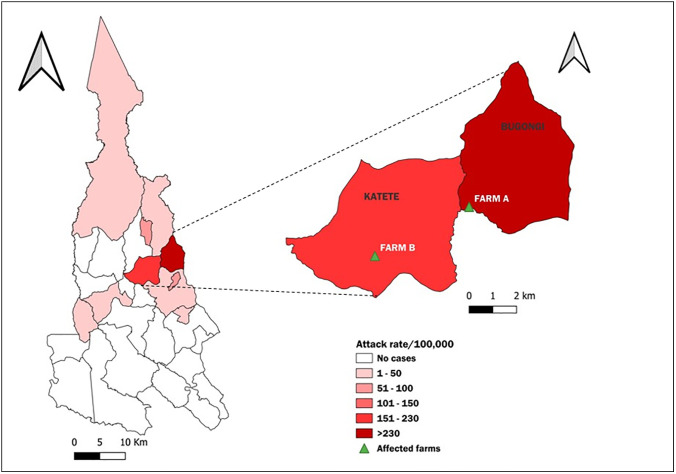
Locations of farms with reported livestock anthrax cases and corresponding human attack rates by sub-county during the anthrax outbreak in Kanungu District, southwestern Uganda, June–November 2024 (map drawn using QGIS browser 3.10.2, based on administrative boundaries provided by the Ministry of Health, Uganda).

Almost 50% of the sub-counties in Kanungu District were affected. Bugongi subcounty was the most affected with an attack rate of 257/100,000, followed by Katete, which had an attack rate of 224/100,000 population. On the map, the green triangles represent the affected farms that were located in Bugongi and Katete (Fig 3). Bugongi hosted Farm A, where the implicated livestock were first introduced, and the livestock didn’t have an animal movement permit. Farm A is a stock farm, and communal grazing takes place here. It also has a stream of water where livestock from different farms drink. Additionally, Bugongi and Katete hosted meat dealers who played a central role in the distribution of contaminated meat. Important to note, livestock that died at the farms were slaughtered at the farms, and the meat was distributed. Through an organized network, the dealers supplied meat to local vendors, nearby villages—greatly amplifying the geographic reach of the outbreak. During this outbreak, a total of 111 animal deaths were registered.

### Hypothesis generation interview findings

Based on the 20 hypothesis-generating interviews, 85% of the respondents reported that they had participated in slaughtering or butchering livestock that had suddenly died, 66% had no or primary education, 60% had consumed meat from contaminated carcasses. In terms of occupation, 20% of the respondents were meat traders, 50% were subsistence farmers, and the remaining 30% had other occupations such as casual laborers or small-scale livestock keepers. We hypothesized that consuming and handling meat from livestock that died suddenly were associated with the June–November 2024 anthrax outbreak in Kanungu District.

### Risk factors for anthrax infection and transmission among cases in Kanungu District, Uganda, June–November, 2024

A total of 270 residents (90 cases vs 180 controls) in Kanungu District were included in the study. Compared to those who did not consume or handle livestock that had suddenly died, contact with meat or products from livestock that had suddenly died (aOR=8.2, 95%CI = 3.8-18) and consuming meat (aOR=5.5, 95%CI = 2.7-11) were associated with higher odds of developing anthrax. Additionally, those who had low education level had higher odds of developing anthrax (aOR=6.0, 95%CI = 2.6-14) compared to those who had attained higher education. Males had higher odds of developing anthrax compared to females (aOR=2.3, 95%CI = 1.03-4.9) (Table 2).

**Table 2:** Factors associated with human anthrax infection, Kanungu District, Uganda, June–November, 2024.

Variable	cOR	95% CI	p-Value	aOR	95% CI	p-Value
**Age group**						
<30	Ref			Ref		
30-50	1.8	(0.80-4.1)	0.153	2.4	(0.83-6.9)	0.108
>50	1.8	(0.87-3.7)	0.116	1.4	(0.55-3.6)	0.474
**Sex**						
Females	Ref			Ref		
Males	2.6	(1.5-4.6)	0.001	2.3	(1.03-4.9)	0.04
**Education level**						
High	Ref			Ref		
Low	3.6	(2.0-6.7)	<0.0001	6	(2.6-14)	<0.0001
**Handled dead animals suddenly**						
No	Ref			Ref		
Yes	14	(7.4-25)	<0.0001	8.2	(3.8-18)	<0.0001
**Consumed suddenly dead animals**						
No	Ref			Ref		
Yes	10	(5.6-19)	<0.0001	5.5	(2.7-11)	<0.0001

cOR: Crude Odds Ratio; aOR: Adjusted Odds Ratio.

### Common reference group analysis for anthrax risk factors

Individuals who ate contaminated meat (aOR=9.0, 95%CI = 3.7-22) and those who had contact with this meat (aOR=17, 95%CI = 5.6-52) had higher odds of infection compared to those who had neither of the exposures. The odds were higher for those who both ate and had contact with contaminated meat (aOR=46, 95%CI = 19–113) (Table 3).

**Table 3:** Common reference group analysis for anthrax risk factors.

Ate meat	Contact only*	Cases (n = 90)	Control (n = 180)	aOR	95% CI
–	–	9	125	Ref	Ref
+	–	20	31	9	3.7-22
–	+	11	9	17	5.6-52
+	+	50	15	46	19-113

* “Contact Only” refers to handling activities detailed in Table 4 (skinning, dissecting, cleaning internal organs, carrying dissected parts). aOR = adjusted odds ratio; CI = confidence interval; Ref = reference category.

### Common reference group analysis for cutaneous anthrax

All forms of handling contaminated meat were associated with anthrax. The odds were the highest among those who participated in multiple handling activities, such as skinning, dissecting, cleaning internal organs, and carrying dissected parts (aOR=44,95%CI = 5.6-354) (Table 4).

**Table 4:** Common reference group analysis for cutaneous anthrax.

Skinning	Dissecting	Cleaned internal organs	Carrying dissected parts	aOR	(95% CI)
–	–	–	–	Ref	Ref
+	–	–	–	12	(2.4-62)
–	+	–	–	8.1	(1.4-45)
–	–	+	–	4	(0.25-66)
–	–	–	+	4.9	(1.9-13)
+	+	+	+	44	(5.6-354)

+: Had contact with contaminated meat; -: Had no contact with contaminated meat.

## Discussion

This outbreak comprised primarily cutaneous cases, followed by gastrointestinal presentations, with a small proportion exhibiting both forms. Almost half of Kanungu District was affected, with an overall case fatality rate of 6.7%. Lower educational attainment (no formal education or only primary schooling) was associated with an increased odds of anthrax infection compared to higher educational attainment (secondary or tertiary). Exposure to carcass handling activities: skinning, dissection, transportation of parts and organ preparation significantly increased the odds of cutaneous anthrax. Consumption of meat from livestock that died suddenly considerably increased the odds of gastrointestinal anthrax. This outbreak was characterized by a predominance of cutaneous over gastrointestinal anthrax cases. This observation is congruent with the global pattern, in which cutaneous anthrax accounts for roughly 85 percent of all human infections [12]. Our findings mirror those from Bangladesh, where most anthrax cases had cutaneous presentations and few gastrointestinal cases were reported, as well as reports from previous anthrax outbreaks in Uganda [25,29–32].

We noted a significant delay in outbreak detection, with human anthrax cases not detected until 83 days, by which time nearly half of Kanungu district was affected. This delay revealed critical gaps in routine veterinary and human surveillance, a low suspicion index among health workers, and weaknesses in the operationalization of the One Health framework at the district level. The prolonged detection period likely contributed to the wide geographic spread and a higher case fatality rate compared to previous anthrax outbreaks in Uganda [32,33]. These findings underscore the need to strengthen sub-national One Health teams to enable integrated surveillance and early detection of public health emergencies. Additionally, targeted capacity-building initiatives are essential to improve the suspicion index among health workers, enabling prompt recognition and reporting of zoonotic events.

We found that lower educational attainment was associated with higher odds of developing anthrax compared to higher education attainment. This association could reflect limited knowledge and awareness of the risks associated with handling and consuming meat from the sudden death of livestock and lower uptake of preventive measures, leading to engagement in high-risk behaviours [1,34,35]. Additionally, individuals with lower levels of formal educational levels are often employed in high-risk occupations such as herding, slaughtering, and informal meat processing, increasing their likelihood of contact with infected livestock or environments where protective measures may be limited [36–38]. Targeted risk communication and health education interventions should prioritize these high-risk groups. However, education level may also function as a proxy for occupation, socioeconomic status, or specific exposure-related behaviors, and the observed association should be interpreted within a broader context.

Case-patients either handled or consumed meat from livestock that had died suddenly. Previous investigations of outbreaks in Uganda have indicated a similar association of anthrax with handling and eating of meat from livestock that died suddenly before slaughter [4,5,32,39]. These findings underscore the urgent need for targeted behavioral change communication to reduce risky practices, strict regulation and monitoring of meat sources, and strengthened community education and surveillance systems. Implementing these measures can help prevent the handling and consumption of potentially infected carcasses, thereby reducing the risk of zoonotic transmission during future outbreaks.

The implicated livestock initially introduced to the affected farms were reported to have been imported from a neighbouring district, underscoring persistent gaps in enforcement of animal movement regulations [34,40]. Furthermore, the involvement of illegal meat dealers in distributing contaminated meat mirrors patterns observed in previous anthrax outbreaks in western Uganda, emphasizing the ongoing risk posed by unregulated meat markets [25]. These findings demonstrate the critical need to enforce bans on uninspected meat, strengthen regulatory oversight of animal movements, and implement targeted community education and risk communication to raise awareness of the dangers associated with consuming unregulated animal products. Collectively, such measures are essential to prevent future anthrax outbreaks and reduce zoonotic transmission.

Based on these findings, several implications for public health emerge. First, the ongoing handling and consumption of meat from livestock that died suddenly exposes persistent gaps in community awareness, safe meat-handling practices, and regulatory enforcement, emphasizing the need to enhance food safety interventions and inspection systems. Second, the involvement of organized meat dealers in sourcing and distributing the carcasses of suddenly deceased livestock poses a significant zoonotic threat that can amplify outbreaks, necessitating strict enforcement against uninspected meat markets and improved surveillance of meat-trading networks. Finally, the link between low formal education and increased anthrax risk highlights the importance of targeted risk communication and health education, particularly for high-risk occupations such as butchering.

### Study limitations

Farmers’ reluctance to report livestock deaths to veterinary teams prevented us from thoroughly investigating animal cases, limiting our ability to quantify the outbreak’s true animal burden. Likewise, relying on healthcare‐seeking records and case definitions may have missed mild or subclinical human infections, some potentially misattributed to routine gastrointestinal illnesses, thus underestimating the outbreak’s actual scope. Although we selected controls from the same villages as the cases to enhance comparability in environmental exposures, the unmatched design and neighborhood-based sampling may not have fully controlled for individual-level differences, potentially resulting in residual confounding. For example, education level may function as a proxy for occupation, socioeconomic status, or specific exposure behaviors related to livestock handling and meat consumption. Additionally, we relied on clinical and epidemiologic linkage for case classification with limited laboratory confirmation, likely due to delayed healthcare seeking and prior antibiotic use that reduced culture yield. Lastly, although environmental samples were collected from outbreak sites, *Bacillus anthracis* was not detected, likely due to heterogeneous contamination, delayed sampling, and limited sample size.

## Conclusions

This anthrax outbreak was associated with the consumption and handling of meat from livestock that died suddenly. This reflected gaps in veterinary inspection, proper carcass management, and regulatory enforcement. Preventing similar outbreaks would require strengthening existing district-level One Health coordination platforms to enhance integrated human–animal surveillance and rapid response to livestock deaths. Reinforcing routine veterinary inspection systems, enforcing hygienic carcass disposal regulations, and strengthening oversight of animal movement and informal meat markets are critical components of this response. Targeted community-based risk communication programs, particularly among herders, butchers, and informal meat traders, could promote early reporting of suspicious livestock deaths and discourage consumption of meat from animals of unknown cause of death. Coupled with routine livestock vaccination campaigns in endemic areas, these programmatic interventions could reduce zoonotic transmission and improve preparedness for future outbreaks.

### Public health actions

We disseminated the findings to the Kanungu District Task Force (DTF) and leaders of the affected communities. Subsequently, the DTF resolved to impose a provisional quarantine on livestock and their products, and we conducted community sensitization about the risks of handling and consuming meat from suddenly dead livestock.

## Supporting information

S1 Fig Location of Kanungu District in Uganda, where the anthrax outbreak occurred, June–November, 2024 (map drawn using QGIS browser 3.10.2, based on administrative boundary shapefiles provided by the Ministry of Health, Uganda). (TIF)

S1 Dataset Dataset of cases and controls included in the anthrax outbreak investigation in Kanungu District, Uganda, June–November 2024. This dataset contains individual-level data for cases and controls included in the study. Variables include demographic characteristics (age, gender, occupation, and education level), exposure history (handling and consumption of animal products), case–control status, geographic location (Bugongi, Katete, and other sub-counties in Kanungu), and temporal variables (year, month, and date of symptom onset). Variables with an underscore (_) represent coded versions of the corresponding categorical variables used for analysis.
(XLS)
