# Supplementary figures and images for: Anthrax outbreak associated with the consumption and handling of carcasses of livestock that suddenly died, Kanungu District, Uganda, June–November 2024
Source: PLoS Negl Trop Dis. 2026 Jul 10;20(7):e0013727. doi: 10.1371/journal.pntd.0013727 (PMC13372227; doi:10.1371/journal.pntd.0013727)

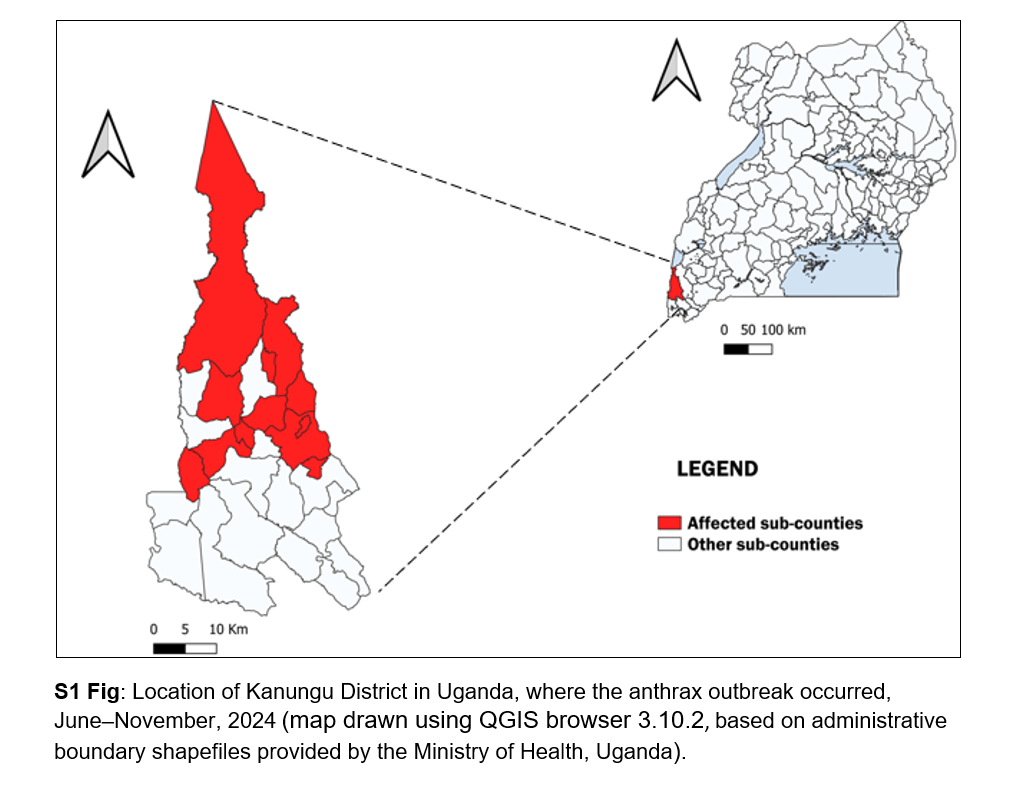

Supplement: S1 Fig — (TIF) [file pntd.0013727.s001.tif]
